# Lack of Investment Value of Insurance

**Published:** 1915-12

**Authors:** 


**Lack of Investment Value of Insurance.** The Med. World, Nov., cites a case in which $6,135 was paid for life insurance of $5,000 in 15 years, the surrender value being $5,683, a loss of $452 and interest which, at 4% in the bank, would have been $1,840. The "dividends" came to $683.46 instead of $1,500 as estimated in advance. We have had a somewhat similar experience, the surrender value being a trifle more than the sum of the premiums but representing a loss of about $1,400, as compared with compound bank interest at 4%. We understand that this is the usual practical experience. We do not in any way underestimate the value of life insurance, especially for a young man without capital. But it should also be understood that $1,000 in a sound savings bank, in the name of the beneficiary, or one's estate, is exactly as good insurance as $1,000 payable on death by the average company.

